# Personality and uveitis

**DOI:** 10.1186/s12348-016-0108-x

**Published:** 2016-10-06

**Authors:** Ankush Kawali, Ringhoo Theresa Jose, Aishwarya, Mathew Kurian, Kushal Kacha, Padmamalini Mahendradas, Rohit Shetty

**Affiliations:** 1 Uveitis and Ocular Immunology Department, Narayana Nethralaya, Bangalore, India; 2 General Ophthalmology Department, Narayana Nethralaya, Bangalore, India; 3 Cataract and Refractive Department, Narayana Nethralaya, Bangalore, India; 4 Narayana Nethralaya, 121/C, Chord road, 1st ‘R’Block, Rajajinagar, Bangalore, 60010 India

**Keywords:** Personality, Uveitis, Immune disease, Behavior

## Abstract

**Background:**

Psycho-immunology is an emerging branch of science which studies the interaction between the brain and the immune system. The purpose of this study is to identify the types of personality factors in patients with non-infectious uveitis and to find its association with a particular uveitic entity if any.

This is a prospective, observational, case-control study of 186 patients with non-infectious uveitis (group A) and controls from general ophthalmology outpatient department (group B). “Global 5/SLOAN” personality questionnaire was used which is based on the five-factor theory of personality which describes personality factors based on the presence or absence of five primary dimensions, viz extroversion, orderliness, emotional stability, accommodation, and intellectual curiosity. Personality factors of patients from groups A and B were compared. History of present illness, clinical diagnosis, details of systemic ailment, and demographic information were collected.

**Results:**

Group A comprised HLA-B27-related uveitis (*n* = 30), uveitis due to sarcoidosis (*n* = 10), Vogt-Koyanagi-Harada syndrome (*n* = 5), sclero-kerato-uveitis due to rheumatoid arthritis (*n* = 5), and idiopathic uveitis in rest. Forty-five patients with uveitis had associated systemic ailment. Uveitis patients (*n* = 56) showed positive personality trait: S (social), C (calm), O (organized), A (accommodative), and I (inquisitive). In contrast, the control group (group B) which mainly comprised patients with non-pathological refractive error and visually insignificant cataract showed more number of negative personality traits (*n* = 62): R (reserved), L (limbic), U (unstructured), E (egocentric), and N (non-curious). This difference between the uveitis and control group was found to be statistically significant (*p* ≤ 0.001). The difference was also statistically significant for O (*p* = 0.008), U (*p* = 0.004), and C (*p* = 0.022) with chi-square test. Calm personality was found to be significantly associated with HLA-B27-related uveitis (*p* = 0.002). N, S, and A traits were seen almost equal in numbers in both the groups. U trait was absent in group A, whereas I trait had negligible presence in group B.

**Conclusions:**

Our finding of an association between organized personality type and uveitis and calm personality and HLA-B27-related uveitis warrants further studies to understand the complex mechanism of psycho-immunology in uveitis.

## Background

Although the etiology and pathogenesis of many diseases have been established in today’s date, there are numerous other conditions especially immune diseases where the exact etiology remains a mystery. One such example is intra-ocular inflammation—uveitis, an immune disorder. Psychosomatic factors and stress hormones have been postulated to be involved in the complicated pathway of inflammation and immune dysregulation. There are various studies reporting associations between antecedent stress and activation of uveitis; however, no causal association can yet be described [1].

The relation between personality and disease has been discussed since the era of Hippocrates. His “Humoral theory” of four fluids—blood, black bile, phlegm, and yellow bile, is well known. He hypothesized that the proper balance between these four fluids is the essence of good health and any imbalance will result in a disease. Personality is a system of parts such as motives, emotions, mental models, and the self, which is organized, develops, and is expressed consistently in a person’s actions [2].

Personality traits often dictate how individuals react to stress and can act either as a protective factor or a catalyst to developing psychological or physical symptoms. There are various theories on personality traits or dimensions. The five-factor theory of personality describes the personality factors based on the presence or absence of five primary dimensions, viz extroversion, emotional stability, orderliness, accommodation, and intellectual curiosity [3].

The link between personality and immunity is an emerging line of study [4, 5]. The relation has even been studied at the molecular and cellular level [6]. There are very few studies conducted to correlate personality and the disease: heart disease and type D personality, central serous chorio-retinopathy (CSCR) and type A personality, open angle glaucoma and hypochondriasis, etc [7–9]. An attempt to study uveitis and personality was made by Lopez et al. in a small series where they assessed type D personality in uveitis patients [10].

To explore link between personality type and predisposition to uveitis, we evaluate personality types in patients with non-infectious uveitis and sclero-kerato-uveitis and compared them with a control group in our population.

## Methods

This study was a prospective, observational, case-control study of 186 patients, 93 with non-infectious uveitis and 93 patients from general ophthalmology clinic (aged 15 to 75 years) presented from April 2015 to June 2016 to our tertiary eye care institute located at Southern India. In this study, we evaluated a middle socioeconomic class of our populations, mainly comprised of Hindu ethnicity who presented to a private, exclusive eye care institution. We analyzed two groups, one with an immune disorder, on multiple topical and systemic medications, and another relatively healthy population on few or no medications. The study was approved by the internal review board and adhered to the Declaration of Helsinki. Informed consent was obtained from all the participants for personality evaluation. Uveitis patients formed group A and the control group formed group B. Diagnosis of uveitis required a comprehensive ocular and systemic history, slit-lamp biomicroscopy, and indirect ophthalmoscopy where needed. Patient with active or resolved uveitis with or without associated systemic disease were included in the study. Acute uveitis cases were first treated for the same and then enrolled into the study after relieving their acute symptoms. Patients diagnosed with HLA-B27 uveitis were diagnosed on the basis on clinical examination and relevant history, regardless of positive or negative HLA-B27 test. Patients diagnosed with traumatic uveitis, postoperative uveitis, infectious uveitis, Fuchs’ uveitis, and masquerade syndromes were excluded from the study. Patients with chronic uncontrolled disease (including uveitis) for more than 3 years were excluded to avoid altered personality secondary to the disease. Patients with other systemic ailments not related to uveitis were excluded except controlled hypertension and diabetes without significant systemic or ocular complications. The same examination protocol was followed for control participants (group B). They were required to have no major ocular or systemic disease. Patients with non-pathological refractive error or mild allergic conjunctivitis or mild form of Meibomian gland dysfunction were allowed to participate in the study. One-eyed patients (regardless of cause) were also excluded from the control group. Patients with mental illness (neurological or psychological) or under treatment for the same, uncooperative patients, patients in agony, and patients with recent ocular or systemic trauma or surgery (within the past 6 months) and physically handicapped patients were excluded from the study (for both groups).

**Fig. 1:**
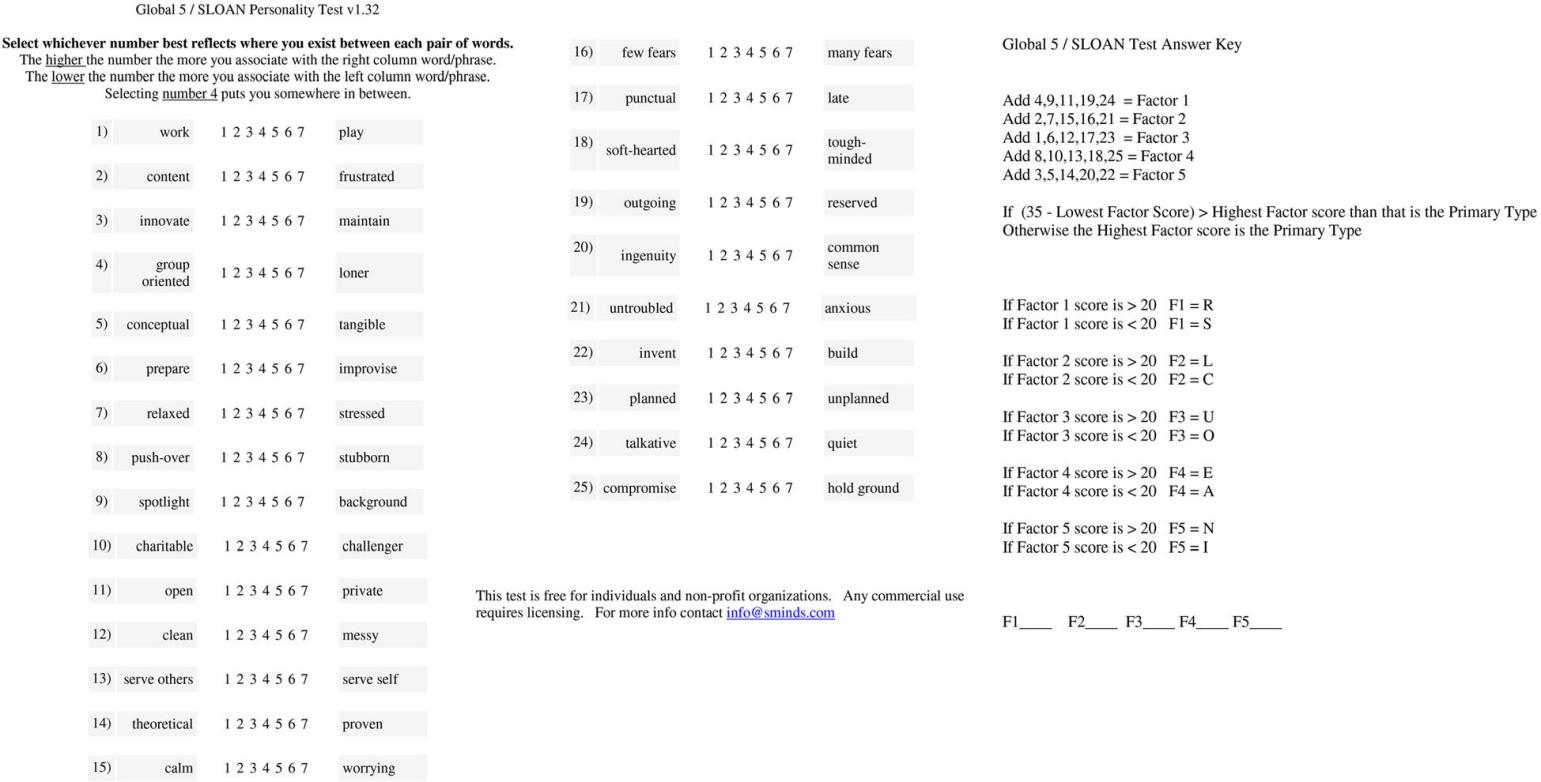
The Global 5/SLOAN personality questionnaire comprise 25 questions to be answered on a 1–7 scale. Primary factor was calculated as per the instructions and was considered as personality type of that particular individual

Patients were asked to answer the “Global 5/SLOAN” personality questionnaire in the outpatient department (OPD) (Fig. 1). Poorly educated patients and patients with poor visual acuity were provided assistance to answer the questionnaire, and patients without knowledge of English language were provided with a questionnaire in their mother tongue (Kannada or Hindi) after due process of translation and back translation and after validation of the translated version by our staff members who knew all three languages. Questionnaire score was then calculated as per the instructions (Fig. 1). And the score “primary type” was taken as the patient’s personality type. Interpretation of primary type was done as per the GLOBAL 5/SLOAN type description. (Available at http://similarminds.com/global5/social.html) Analysis of personality zones (32 SLOAN types) such as SCOAN, SCOAI, RCOAI, and RCOAN was limited due to the small sample size of our study.

Patient’s demographic data, history of present illness, and systemic history were noted. Disease severity and duration of disease were ignored as the primary aim of the study was to find personality type commonly seen in uveitis patients and not the effect of the disease on psychopathology.

Study group (group A) and control group (group B) were compared using chi-square test and Student’s *t* test. Participants meeting inclusion-exclusion criteria were selected during their OPD visit. Post power calculation was done. Sample sizes of 93 in group A and 93 in group B achieved 80 % power to detect a difference between the group proportions of 0.1500. The proportion of calm in group A is assumed to be 0.1080 under the null hypothesis and 0.2580 under the alternative hypothesis. The proportion of calm in group B is 0.1080. The test statistic used is the two-sided *Z* test with pooled variance. The significance level of the test was targeted at 0.0500.

## Results

The study group (group A) with 93 patients and equal number of control group (group B) was sex matched (M:F = 53:40) and had minor variation with respect to age (Table 1). Socioeconomic status was comparable in both the groups. Hindus comprised the largest ethnic portion of both the groups. Five percent of the study population required assistance (five patients in the study group and three patients in the control group). Twenty-eight patients opted for the Kannada version of the questionnaire (21 in group A and 7 in group B) and five patient opted for the Hindi translation of the questionnaire (3 in group A and 2 in group B).

**Table 1:** Characteristics of cases and controls

		Cases (*N* = 93)		Controls (*N* = 93)		*P* value
Gender	M: F	53:40		53:40		1.000^a^
Age groups (years)	10–20	7	7.5 %	9	9.7 %	0.835^a^
	21–30	22	23.7 %	23	24.7 %	
	31–40	25	26.9 %	25	26.9 %	
	41–50	15	16.1 %	19	20.4 %	
	51–60	14	15.1 %	11	11.8 %	
	61–75	10	10.8 %	6	6.5 %	
Age (years)	Mean ± SD	39.1 ± 14.354		35.6 ± 11.996		0.071^b^
	(Min–Max)	12–72		18–65		
Ethnicity	Hindus	87	93.5 %	88	94.6 %	0.290^a^
	Muslim	4	4.3 %	1	1.1 %	
	Christian	2	2.2 %	4	4.3 %	
Positive personality types	S, C, O, A, I	56	60.2 %	31	33.3 %	<0.001^a^
Negative personality types	R, L, U, E, N	37	39.8 %	62	66.7 %	

*S* sociable, *C* calm, *O* organized, *A* accommodative, *I* inquisitive, *R* reserved, *L* limbic, *U* unstructured, *E* egocentric, *N* non-curious, *M* male, *F* female

^a^Chi-square test

^b^Student’s *t* test

In group A, the spectrum of uveitis was as follows: anterior uveitis (*n* = 43), anterior with intermediate uveitis (*n* = 13), intermediate uveitis (*n* = 4), posterior uveitis (*n* = 12), panuveitis (*n* = 14), and sclero-kerato-uveitis (*n* = 7). Diagnosis of HLA-B27-related uveitis was most common in group A. Thirty patients were clinically diagnosed with HLA-B27-associated uveitis, and 19 turn out to be positive for the test (HLA-B27). Ten patients were diagnosed with sarcoidosis-related uveitis, five patients had Vogt-Koyanagi-Harada syndrome, and five patients had rheumatoid arthritis. In the rest of the patients, etiological diagnosis was idiopathic or uncertain but infectious etiologies were ruled out in all. Forty-five patients had systemic association to their uveitis. The spectrum of systemic disease was as follows: ankylosing spondylitis (*n* = 9), rheumatoid arthritis (*n* = 5), cutaneous sarcoidosis (*n* = 2), pulmonary sarcoidosis (*n* = 3), non-specific arthritis (*n* = 20), and interstitial lung disease, tubulo-interstitial nephritis, psoriasis (*n* = 2 each). Thirty-seven patients were on immunomodulatory treatment (IMT) for their uveitis. IMT mainly comprised methotrexate and azathioprine. Patients had received oral and/or topical steroids or steroid injections during active inflammation. In ten patients, better eye vision ranged from 20/40 to 20/80 while others had better eye vision of 20/20 or 20/30.

In group B, 87 % of patients had non-pathological refractive error. Nine patients had mild allergic conjunctivitis and four had a mild form of Meibomian gland dysfunction (similar occurrence was observed in group A). No other ocular or systemic morbidity was present in group B. Diabetic and hypertensive status did not differ significantly in both groups.

**Fig. 2:**
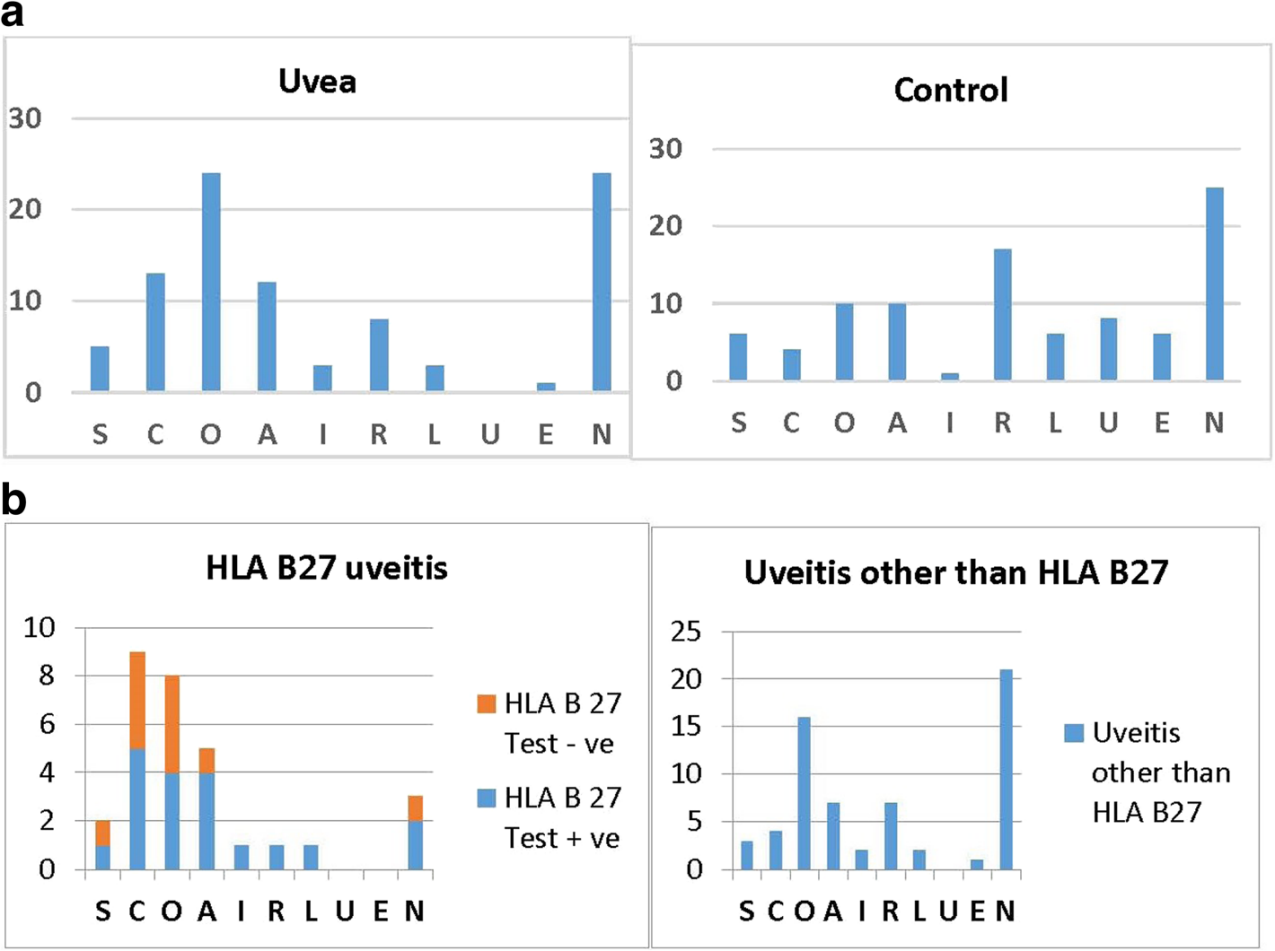
**a** Graphical representation of personalities in the uveitis group versus the control group. Note the almost equal occurrence of *S*, *A*, and *N* traits in both groups, the absence of trait *U* in the uveitis group, and the negligible presence of *I* in the control group. Note the *high bar* of trait *O* in the uveitis group in comparison with the control, which is statistically significant. **b** Graphical representation of personalities in HLA-B27-related uveitis and other uveitic entities. *Orange bar* represents patients tested negative for HLA-B27. Note the high occurrence of trait *C* in HLA-B27-related uveitis

The pattern of primary type of personality in group A and group B was as shown in Fig. 2 and Table 1. Group A patients (*n* = 56) showed positive personality types: S (social), C (calm), O (organized), A (accommodative), and I (inquisitive), and 37 had negative personality type: R (reserved), L (limbic), U (unstructured), E (egocentric), and N (non-curious). O and N traits were seen more commonly. No patient had unstructured (U) personality trait. C, O, and A personality traits were found frequently in HLA-B27 uveitis patients but calm personality trait was found statistically significant compared to other uveitis patients in group A (*p* = 0.002). In contrast to group A, group B showed the presence of more negative personality types. Positive personality traits (S, C, O, A, I) were seen in 31 whereas negative traits (R, L, U, E, N) were seen in 62 participants in group B (Fig. 2). The I trait was least found in group B. This difference of positive and negative personality traits was statistically significant between both groups (*p* ≤ 0.001).

N and A traits were seen almost in equal numbers in both groups. The differences between group A and group B were statistically significant for O (*p* = 0.008), U (*p* = 0.004), and C (*p* = 0.022) on chi-square test.

## Discussion

Our study is the first of its kind, which evaluated various personality types in uveitis patients. Uveitis patients showed different personality types when compared to the control group.

Organized personality, an element of orderliness, which was statistically significant in uveitis patients, describes a person as “more controlled than random, more logical than abstract, more grounded than in the clouds, overachiever, motivated, mature, hard worker and punctual.” This trait has many positive attributes but are also prone to anxiety and stress when circumstances are perceived as not under one’s control such as illness (available at http://similarminds.com/global5/organized.html).

Personality types in patients with non-inflammatory chronic or recurrent ocular diseases were not evaluated in this study, and this was a drawback. It is possible that patients with chronic ocular diseases other than uveitis may also show similar personality pattern to that of uveitis patients. A study in open-angle glaucoma patients showed that their patients had higher hypochondriasis and hysteria [9]. In contrast, our study noted predominance of calm personality than limbic in the uveitis group.

Various studies reported the association between anxiety, depression, and ankylosing spondylitis [11–13]. Martindale et al. found that in ankylosing spondylitis (with or without associated uveitis), disease activity scores correlate significantly with psychological statuses like anxiety and depression [11]. Anxiety and depression can develop as a result of the disease itself. Our aim in this study was different, we tried to investigate an association between personality type and susceptibility to uveitis and we found that HLA-B27-related uveitis patients (which also included patients with ankylosing spondylitis, *n* = 9) had calm personality compared to other uveitic entities (Fig. 2).

Both groups showed almost equal occurrence of sociable, accommodative, and non-curious personality traits (*p* = 0.756, 0.650, 0.868, respectively). The lack of unstructured trait in group A and its preponderance in group B is a further validation of our findings as orderliness and unstructured traits lie on opposite ends of the personality dimension.

The relationship between personality trait and immune system has been recently studied by many researchers. In ocular diseases, Ventura has discussed how stress is mediated through sympathetic adrenomedullary and hypothalamic pituitary axis activation with shifts in immunity. He postulates that psycho-neuro-immunology would provide meaningful answers to etiology and progression of ocular diseases like uveitis, glaucoma, keratitis sicca, and wound healing [14]. Marsland and collaborators reported that trait negative affect has less protective immune response [15]. Eric Jaffe’s review mentions that hostility produces more inflammatory cytokines, although not every study has found a significant link [16]. Vedhara et al. have noted that extraversion is associated with increased expression of pro-inflammatory genes and conscientiousness was associated with reduced expression of pro-inflammatory genes [17]. Link between introversion and vulnerability to infections has been studied at the genetic level by documenting immune response gene polymorphism in introverts [18]. This suggests that biological immune response antagonizes behavioral immune response (social avoidance) [17]. Lopez et al. in their series showed that uveitis patients had type D personality (negative affectivity and social inhibition) when compared with a control, although not statistically significant [10]. In contrast, we found almost equal occurrence of sociable trait in both groups (*p* = 0.756). Moreover, control population was found to score high for reserve personality trait (*p* = 0.053). Association of organized personality trait and uveitis, as noted in our study, needs to be studied further in uveitis patients in a larger study as well as in other immune diseases to validate its association. The positive attributes of being controlled and orderly may have good prognostic effect on treatment adherence measures while the anxiety generated by a uveitis relapse maybe compounded by feelings of losing control and descending into chaos. Gupta et al. have described their patients of rheumatoid arthritis as “leading quiet lives” [19]. Similarly, we noted an association of calm personality type and HLA-B27-related uveitis.

A double-blind controlled trial has shown that psychological interventions such as cognitive behavior therapy can improve biological markers of disease activity (ESR, CRP, RA factor) in rheumatoid arthritis patients [20]. One can hypothesize that, if personality can be altered, it could serve as an adjunctive therapeutic option to the standard of care treatment especially when uveitis is not under control with maximum possible medications. HLA-B27-related uveitis is notorious to form posterior synechiae rapidly, and we know increased sympathetic discharge dilates the pupil. Therefore, if a calm personality of a patient with HLA-B27-related uveitis changed to aggressive (one with more sympathetic discharge), will it help in breaking posterior synechiae is an evolving research question.

Although we discovered a link between personality and uveitis, our study was limited by several biases like a small sample size, translation of the questionnaire to the local language (*n* = 33), lack of interview by a psychologist, and absence of a psycho-therapeutic intervention. Secondly, we could not evaluate personality separately for each age group and for male and female separately due to the small sample size. Being a small study, it was not possible to evaluate various different etiologies of non-infectious uveitis (except HLA-B27-related uveitis) and their personality type separately. Different types of non-infectious uveitis and their personality type will be the forthcoming of this project.

## Conclusions

In conclusion, our finding of an association between organized personality type and uveitis and an association between calm personality and HLA-B27-related uveitis warrants larger studies to further understand the complex mechanism of psycho-immunology and uveitis, as this may contribute to novel therapeutic approaches in the field of uveitis.

## Abbreviations

A: accommodative
C: calm
CSCR: Central serous chorio-retinopathy
E: egocentric
I: inquisitive
IMT: immunomodulatory treatment
L: limbic
N: non-curious
O: organized
R: reserved
S: social
U: unstructured
